# Graded exercise test with or without load carriage similarly measures maximal oxygen uptake in young males and females

**DOI:** 10.1371/journal.pone.0246303

**Published:** 2021-02-01

**Authors:** Zhenhuan Wang, Muhammed M. Atakan, Xu Yan, Hüseyin H. Turnagöl, Honglei Duan, Li Peng

**Affiliations:** 1 Key Lab of General Administration of Sport, Southwest University, Chongqing, China; 2 Institute for Health and Sport, Victoria University, Footscray, Melbourne, Australia; 3 Division of Nutrition and Metabolism in Exercise, Faculty of Sport Sciences, Hacettepe University, Ankara, Turkey; 4 College of Health and Biomedicine, Victoria University, Melbourne, Australia; University of Pavia, ITALY

## Abstract

The aim of this was to compare the effects of the graded exercise test (GXT) with or without load carriage on maximal oxygen uptake ($\dot{V}O_{2\max}$) heart rate (HR), and expired ventilation ($\dot{V}E$) and blood lactate in young healthy males and females. The study included ten females (age:20.2±0.7 yrs) and ten males (age:19.5±0.7 yrs) who performed the modified Bruce protocol at five load conditions; unloaded, 5, 10, 15, and 20% of body weight (BW) (kg). All the tests were performed in random order, at least 48 hours apart. During the GXTs, HR, $\dot{V}O_{2\max}$, $\dot{V}E$, workload and test duration were recorded and blood lactate concentration was measured before and immediately after the GXTs. $\dot{V}O_{2\max}$ remained unchanged during the GXTs in load and unloaded conditions for both sexes (p>0.05). Test duration was significantly less in females during the GXT with 15% BW (15.9±0.51 min vs. 18.1±1.14 min; p = 0.014) and 20% BW load carriage (15.2±0.75 min vs. 18.1±1.14 min; p = 0.020), compared to the unloaded GXT. Males showed significant decrease in the test duration during the GXT with load 15% BW (20.5±0.53 min vs. 22.8±0.61 min; p = 0.047) and with 20% BW (19.6±0.42 min vs. 22.8±0.71 min; p = 0.004), compared to the GXT with 5% BW. $\dot{V}E$ statistically decreased in female subjects only at 15% BW compared to 20% BW (15% BW = 77.9 ± 10.5 L/min vs. 15% BW = 72.0 ± 10.9 L/min; p = 0.045). There was no difference observed in maximal HR and blood lactate concentration between the GXTs in load and unloaded conditions. This study indicates that no matter the load % used during the GXT, $\dot{V}O_{2\max}$, but not total exercise time, remains the same in young males and females.

## Introduction

Maximal oxygen uptake ($\dot{V}O_{2\max}$) is the maximum integrated capacity of the pulmonary, cardiovascular and muscular systems to uptake, transport and utilize oxygen, respectively [1]. $\dot{V}O_{2\max}$ is typically measured as a powerful predictor of mortality for cardiovascular disease [2] and for exercise prescription [3]. For this reason $\dot{V}O_{2\max}$ test has been a cornerstone in clinical and applied physiology and has been widely used in different populations including athletes, people with several pathological conditions [4–7]. Exercise physiologists have been in a search for an optimal protocol suitable for the entire spectrum of fitness abilities and testing goals to determine $\dot{V}O_{2\max}$ and established some methods by which $\dot{V}O_{2\max}$ can be measured [8–10]. The most common ones of these methods that consist of an incremental exercise test are the graded exercise test (GXT), self-paced $\dot{V}O_{2\max}$ test and progressive test protocols on treadmill or cycle ergometer [11, 12]. While the treadmill is the most widely used modality due to familiarity with upright locomotion and greater muscle mass and muscle recruitment [13], cycling protocols provides an opportunity to test individuals with coordination or orthopedic limitations [11].

Running on a treadmill requires maintenance of balance and motion of the arms and legs relative to the body, which are expected to contribute to energy expenditure. Walking with an extra load on a smooth treadmill surface at fixed speed [14, 15] expends considerably more effort and increases metabolic energy expenditure, step width variability, muscle recruitment and demands on balance [14, 15]. These factors are associated with increased the overall energy cost of running measured with $\dot{V}O_{2\max}$ [16]. Heavy load carriage weighing to 35-kg during an acute exercise [17], 31.4-kg during walking on downhill and uphill surfaces [18] or 45-kg thoracic loading during GXT to exhaustion on treadmill [19] leads to changes in physiological demand [17–19]. Taylor et al. [20] assessed the impacts of wearing the personal protective equipment including protective clothing, helmet, breathing apparatus and boots upon performance in healthy male and female adults and reported reduced exercise tolerance by 56% during steady-state walking on a treadmill, with the ambulatory oxygen uptake reserve being 31% lower [20]. Grenier et al. demonstrated that 3-min of walking at constant speed (4 km/h) on treadmill with load weighing to 27% and 46% body weight (BW) significant changed walking mechanics and energetics in ten middle-aged male subjects [21]. Carrying these type of backpacks during the GXT or incremental exercise tests performed on treadmill was also reported to reduce exercise performance and test duration without changing peak minute ventilation and oxygen uptake [22], suggesting that weight of the external load carriage reduced treadmill performance without affecting the cardiopulmonary system. Besides, the modified Balke treadmill exercise test with 20.4 kg backpack was shown to decrease peak oxygen uptake ($\dot{V}O_{2\mathrm{peak}}$) in physically active males and females, when compared to the unloaded condition [23], which might be partially explained by breathing pattern at peak exercise which is more rapid and shallow, compared to the unloaded condition, suggesting increased wasted ventilation. A study by Phillips et al. [19] has reported that progressive increases in thoracic load (45-kg) during a GXT reduced the absolute O_2_ uptake, minute ventilation, power output, and test duration [19]. However, there is no study we are aware of that has been done thus far to show if the GXT performed in load and unloaded condition can similarly measure the $\dot{V}O_{2\max}$ in healthy males and females. Moreover, despite a growing body of work investigating ventilatory, heart rate (HR), or $\dot{V}O_{2\max}$ responses to exercise with load carriage, the majority of the published studies have involved male subjects [17–19, 24, 25], with limited research investigating physiological responses in females [22, 23, 26–28]. Therefore, the results can not be generalized to females, who are, on average, smaller in mass, stature and functional capacity [29], smaller lungs, smaller airways, increased work of breathing during exercise and decreased aerobic power [30], compared to height-matched males. In this context, the main aim of this study was to compare the effects of the unloaded GXT with the GXTs performed at four different load conditions; 5%, 10%, 15%, and 20% BW on $\dot{V}O_{2\max}$, HR, and expired ventilation ($\dot{V}E$) and blood lactate, test duration in healthy males and females. We hypothesized that the effects of the GXTs with or without load carriage on $\dot{V}O_{2\max}$, HR, and $\dot{V}E$ and blood lactate would be similar in young males and females and the GXTs performed with load carriage would reduce the test duration for both sexes.

## Materials and methods

### Study design

The study was approved by the Human Research Ethical Committee of the Affiliated Hospital of Southwest University, Chongqing, China (Approval no: SWU-TY-2018-03). This study was a repeated-measures design, conducted at the Key Lab of General Administration of Sport, Southwest University. The study protocol included the GXTs to exhaustion performed at five different load carriages; unloaded, 5, 10, 15 and 20% BW loads. In order to avoid circadian variations on the outcomes, all the GXTs were performed in random order at the same period of the day (12:00 noon– 3:00 PM), in controlled temperature conditions (21±2°C) and relative air humidity (about 60%). To minimize any diet-induced variability in the GXTs, the subjects were asked to record food and fluid consumption before the first GXT whereby they replicated the same diet before the other GXTs. The subjects were instructed to arrive at the laboratory in a rested state, at least 3 h postprandial, and to avoid strenuous exercise in the 24 h preceding each testing session. Each subject was asked to refrain from caffeine and alcohol 24 h before each test. The subjects were required to report to the laboratory on 5 occasions over a 4- to 5-wk period, and all the tests were interspersed with at least 48-h recovery.

### Subjects

The characteristics of the subjects are presented in Table 1. A total of 10 males and 10 female volunteered to participate in this study. The subjects participated in exercise at a recreational level and had previously participated in studies where treadmill exercise testing similar to the current study was applied. All subjects were required to give their written, informed consent before the commencement of the study, once the experimental procedures, associated risks, and potential benefits of participation had been explained. The GXTs data of one subject who had suffered a minor joint injury during the unloaded GXT were excluded from the study. Accordingly, data from 19 subjects (male = 9/female = 10) were included in the final analysis. Inclusion criteria were: recreationally active males and females, non-smokers, non-alcoholic, free of acute or chronic disease, aged 20–30 years, not participating in any systematic training program, including endurance, sprint, and resistance. The subjects were excluded if they had a known exercise-limiting cardiovascular, respiratory or metabolic illness or on any medication known to affect metabolism. All subjects completed the International Physical Activity Questionnaire.

**Table 1:** Subjects characteristics.

	Male	Female
**Age (yrs)**	19.5 ± 0.7	20.2 ± 0.7
**Height (cm)**	172.0 ± 6.6	164.7 ± 1.8
**BW (kg)**	65.1 ± 7.8	56.4 ± 5.5
**BMI (kg/m**^**2**^**)**	21.4 ± 1.4	20.3 ± 1.7
$\dot{V}O_{2\max}$ **(ml/kg/min)**	54.3 ± 7.2	41.3 ± 8.5
**Resting HR (beat/min)**	74.6 ± 6.8	78.1 ± 8.3
**Maximal HR (beat/min)**	195.5 ± 7.5	189.9 ± 17.4

BW, body weight; BMI, body mass index; $\dot{V}O_{2\max}$, maximal oxygen uptake; HR, heart rate; values are means ± SD for 19 (male n = 9, female n = 10).

### Graded exercise tests

On the first day when the first GXT was performed, height was measured to the nearest 0.1 cm using a stadiometer (InBody BSM370, Biospace, Seoul, Korea). BW was measured to the nearest 0.1 kg using a scale (InBody 3.0, Biospace, Seoul, Korea) on the GXTs days both for the $\dot{V}O_{2\max}$/kg values of the different GXTs and to ensure that the backpacks with correct weight were provided for each subject. Body mass index (BMI) scores were calculated [weight (kg)/height (m)^2^]. All subjects completed randomly ordered the GXTs in load and unloaded conditions. Resting HR was measured for a 3-minute period in a supine position in a calm environment (in the morning) after a 10-minute period of supine resting and was defined as the lowest one-minute average during the sampling period. Since the Bruce protocol can lead to ambulation difficulties and the large increments in workload between stages can likely lead to premature discontinuation of exercise test and an underestimation of our participants true workload capacity [31], the modified Bruce protocol that starts off at the same speed but with an initial grade of 0%, has a lighter initial increment, was used to assess $\dot{V}O_{2\max}$ of the subjects with the unloaded, 5, 10, 15 and 20% BW backpacks carried on the mid-back region [32]. The mid-back region was specifically chosen as it was shown to be associated with a lower energy cost than most other forms of load carriage [33, 34]. Following 4-min of quiet standing, the subjects began walking at 2.7 km/h at 0% incline, stage 2 is at 2.7 km/h at 5% incline, and then stage 3 was the start (Stage 1) of the Bruce protocol consisting of further increases in grade and speed continue, at 3-min intervals, until the subject reaches volitional exhaustion (Run-7410, Italy) (S1 Table) [35]. During the GXTs, HR (Polar-T3, Kempele, Finland), oxygen uptake, $\dot{V}E$ (L·min^-1^) throughout the tests were recorded using Breath-by-Breath technology (Metalyzer II-R2, CORTEX, Germany) and the data collected and exercise duration were automatically recorded by the software. The distance covered during the GXTs was recorded. During the test, the subjects were supported by verbal encouragement to continue until volitional exhaustion. When the subjects could no longer continue, they were instructed to grab the handrail of the treadmill, at which time the test was terminated. The time average of the last 30 seconds was used to determine the $\dot{V}O_{2\max}$ value as suggested by Robergs et al. [36] who recommended no longer than a 30-second time average. The exercise test was valid when at least two of the following criteria were met: (a) plateauing of $\dot{V}O_{2}$ while increasing work rate, (b) a respiratory exchange ratio (RER) greater than 1.1, and (c) an HR within 10 beats/min of the predicted maximum (220—age beats/min), (d) ≥18 from rating of perceived exertion scale_6-20_ [37, 38]. Besides, it is known that a plateau in $\dot{V}O_{2}$ is a necessary consequence of an incremental exercise test and the best criteria for establishing $\dot{V}O_{2\max}$ [39], and that a failure to attain a $\dot{V}O_{2}$ plateau is likely due to “insufficient effort” from the subject and result in a $\dot{V}O_{2\mathrm{peak}}$ that is not truly the individual’s maximum capacity [40]. Therefore, the subjects were thoroughly informed about the testing procedure and encouraged not to interrupt the GXTs before reaching the $\dot{V}O_{2}$ plateau criterion verified based on visual inspection of the recorded data on the computer screen. Accordingly, most of the GXTs (89%) performed were completed once the plateaus in $\dot{V}O_{2}$ were reached, whilst the GXTs during which the plateau in $\dot{V}O_{2}$ was not reached (11%) were terminated once the subjects met at least two of the other three criteria.

The test continuously monitored on the computer screen throughout the tests to supervise the physiological response to the GXTs. The $\dot{V}O_{2\max}$ presented in Table 1 was the result of the $\dot{V}O_{2\max}$ value achieved at the end of the unloaded GXT. Prior to each test, air and periodic volume calibrations were made according to procedures prescribed by the manufacturer. Finger prick blood samples were collected using Lactate Scout (EKF, Germany) before and immediately after the GXTs to measure blood lactate concentration.

### Statistical analysis

The sample size was calculated based on repeated measures ANOVA with within factors using the statistical program G*Power software [41], which showed that 10 subjects per group were needed, with an effect size f of 0.30, an α error probability of 0.05, a power of 0.85, a correlation among repeated measures of 0.5 and a nonsphericity correction ε of 1. Cohen’s effect sizes (d) were classified as small (0.20), moderate (0.60), and large (0.80) and were calculated from data on the means, the number of subjects and the pooled standard deviations (SD) [42]. Shapiro–Wilk test was performed to determine if the data set was well-modelled by a normal distribution for male and female subjects [43] and all were normally distributed. Repeated measures ANOVA performed separately for male and female subjects were used to detect any differences in the measured variables during the GXTs. If a significant difference was found, the Bonferroni correction test was applied to determine where the difference resided. The p-value of the Mauchly's sphericity test was checked to see if the assumptions were violated and accordingly the p-value of the sphericity assumed (if p>0.05) or the p-value of the Greenhouse-Geisser (if p<0.05) was considered. Independent sample *t*-test was also used to determine if differences between males and females in terms of test duration. All statistical analyses were performed using IBM SPSS Statistics for Windows, Version 21.0 (Armonk, NY: IBM Corp, USA). Significance was set at p<0.05. All data are presented as mean ± SD.

## Results

**Fig 1:**
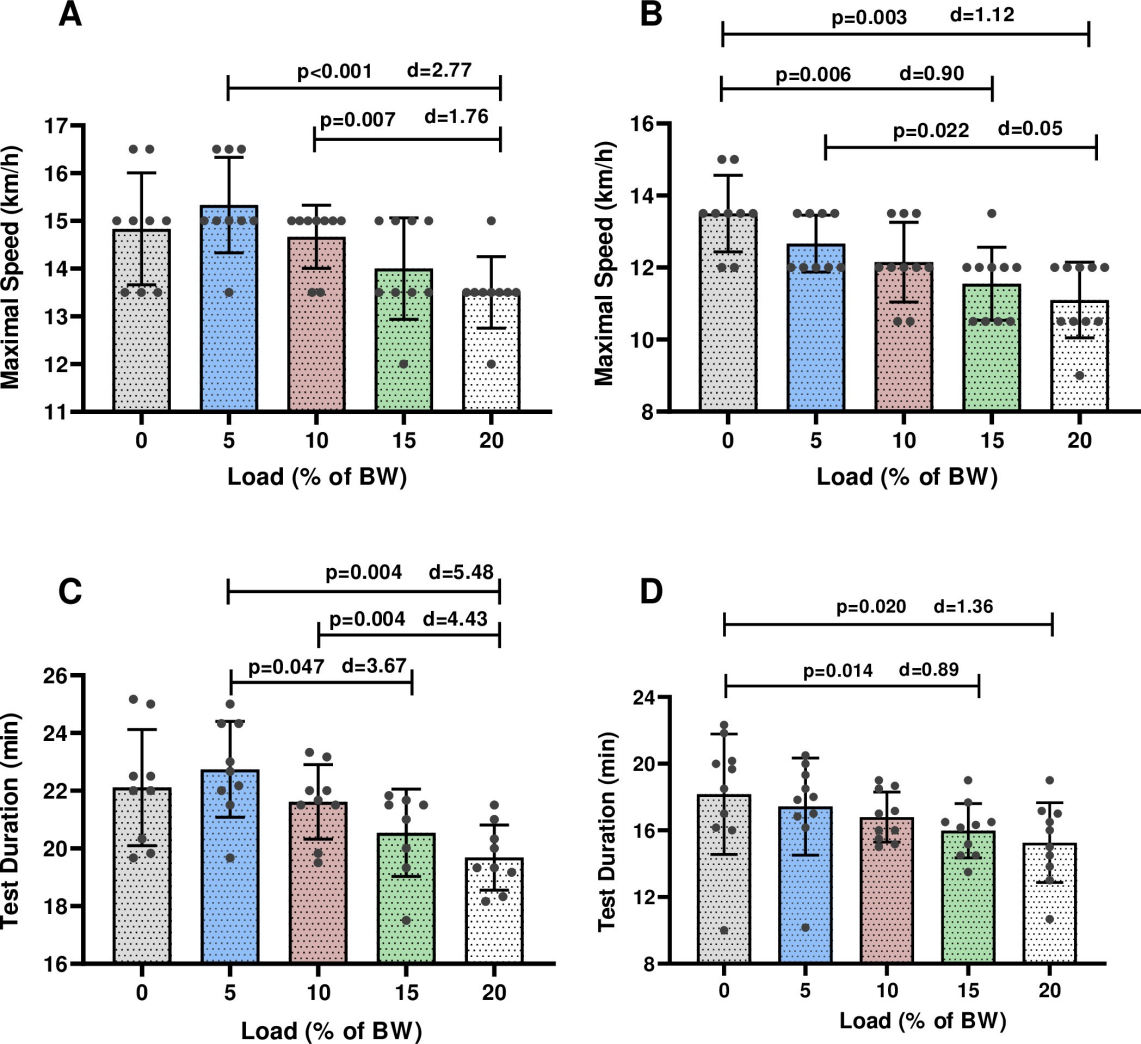
Mean and standard deviation of maximal speed and test duration at different loads in males (A-C) and females (B-D) during the GXTs. p = significant level; d = Cohen’s effect sizes.

There was a gradual decrease observed in maximal running speed (km/h) as the load increased for both sexes. Females and males reached maximal running speed (female = 13.50 km/hour—19.07 min) and maximal test duration (min) (male = 15.30 km/h—22.6 min) at the end of the unloaded GXT and the GXT with 5% BW load condition, respectively (Fig 1).

Males reached lower maximal running speed at the end of the GXT with 20% BW (13.5 ± 0.75 km/h), compared to the GXTs with 5% (15.3 ± 1.0 km/h; p<0.001) (d = 2.77) and 10% BW (14.6 ± 0.66 km/h; p = 0.007) (d = 1.76) (Fig 1A).

The maximal running speed reached at the end of the unloaded GXT was significanly higher in females, compared to the GXT with 15% BW (13.5 ± 1.06 km/h vs. 11.1 ± 1.05 km/h min; p = 0.006) (d = -0.90) and the GXT with 20% BW (13.5 ± 1.06 km/h vs. 11.1 ± 1.05 km/h min; p = 0.003) (d = -1.12) (Fig 1B). There was also significant difference in the maximal running speed between the GXT with 5% BW (12.6 ± 0.79 km/h) and 20% (11.1 ± 1.05 km/h) (p = 0.022) (d = 0.05) in females (Fig 1B).

Males showed significant decrease in the test duration during the GXT with load 15% BW (20.5 ± 1.51 min vs. 22.8 ± 1.6 min; p = 0.047) (d = 3.67) and with 20% BW (19.6 ± 1.1 min vs. 22.8 ± 1.6 min; p = 0.004) (d = 5.48), compared to the GXT with 5% BW (Fig 1C). Test duration at the GXT with 20% BW was less in males compared to the GXT with 10% BW (19.6 ± 1.1 min vs. 21.6 ± 1.2 min; p = 0.004) (d = 4.43) (Fig 1C). Test duration of the GXTs was significantly less in females during the GXT with load 15% BW (15.9 ± 1.6 min vs. 18.1 ± 3.6 min; p = 0.014) (d = 0.89) and with 20% BW (15.2 ± 2.2 min vs. 18.1 ± 3.6 min; p = 0.020) (d = 1.36) than the unloaded GXT (Fig 1D).

**Fig 2:**
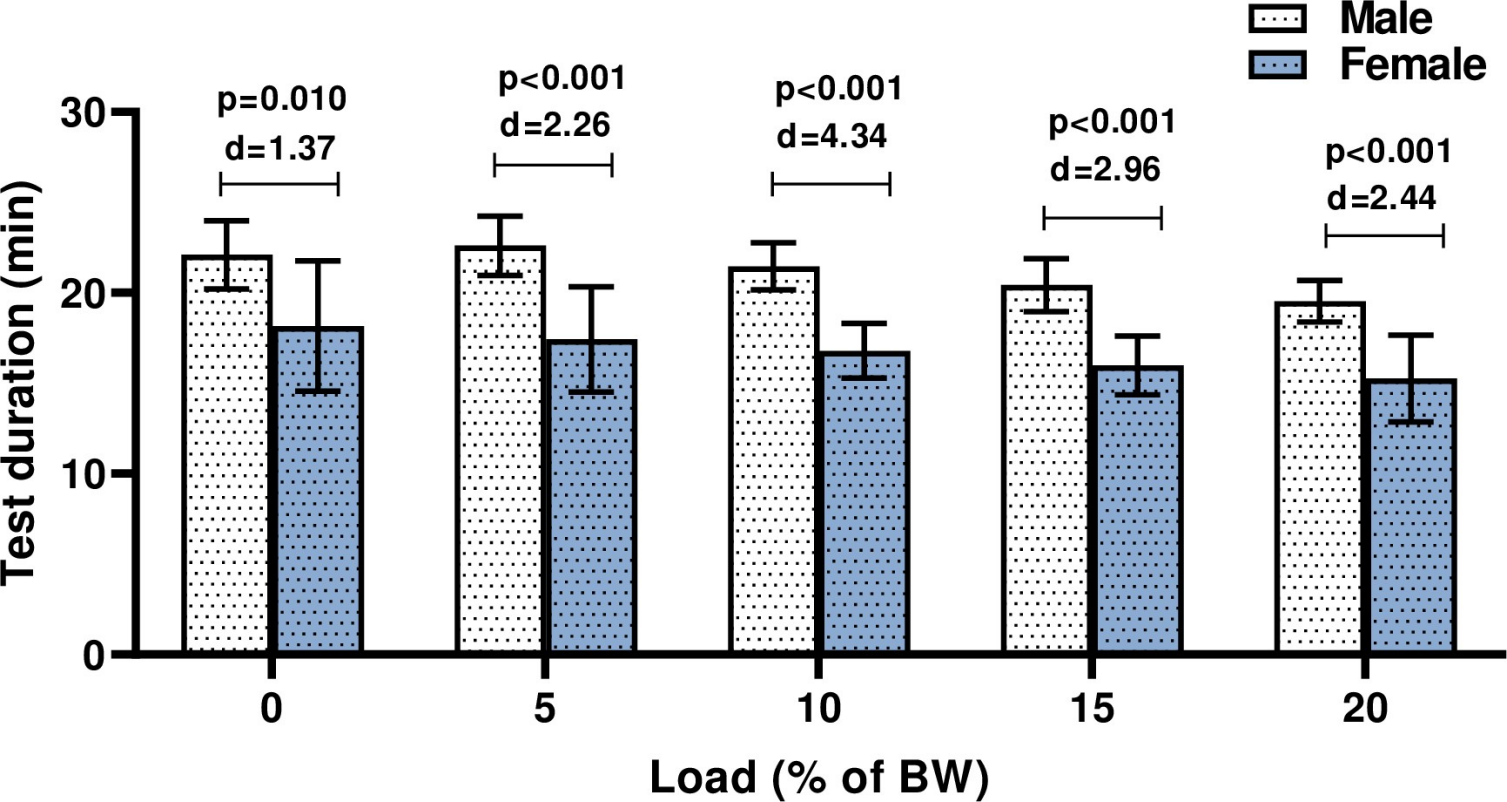
Comparing the test duration of the GXTs in male and female subjects. p = significant level; d = Cohen’s effect sizes; BW, body weight.

Compared to female, males subjects reached higher test duration during the GXTs in unloaded (22.1 ± 2.0 min vs. 18.1 ± 3.6 min; p = 0.010 (d = 1.37) and load conditions (5% BW = 22.7 ± 1.6 min vs. 17.4 ± 2.9 min; p<0.001) (d = 2.26) (10% BW = 22.6 ± 1.2 min vs. 16.7 ± 1.5 min; p<0.001 (d = 4.34) (15% BW = 20.5 ± 1.5 min vs. 15.9 ± 1.6 min; p<0.001 (d = 2.96) (20% BW = 19.6 ± 1.1 min vs. 15.2 ± 2.3 min; p<0.001 (d = 2.44) (Fig 2).

**Fig 3:**
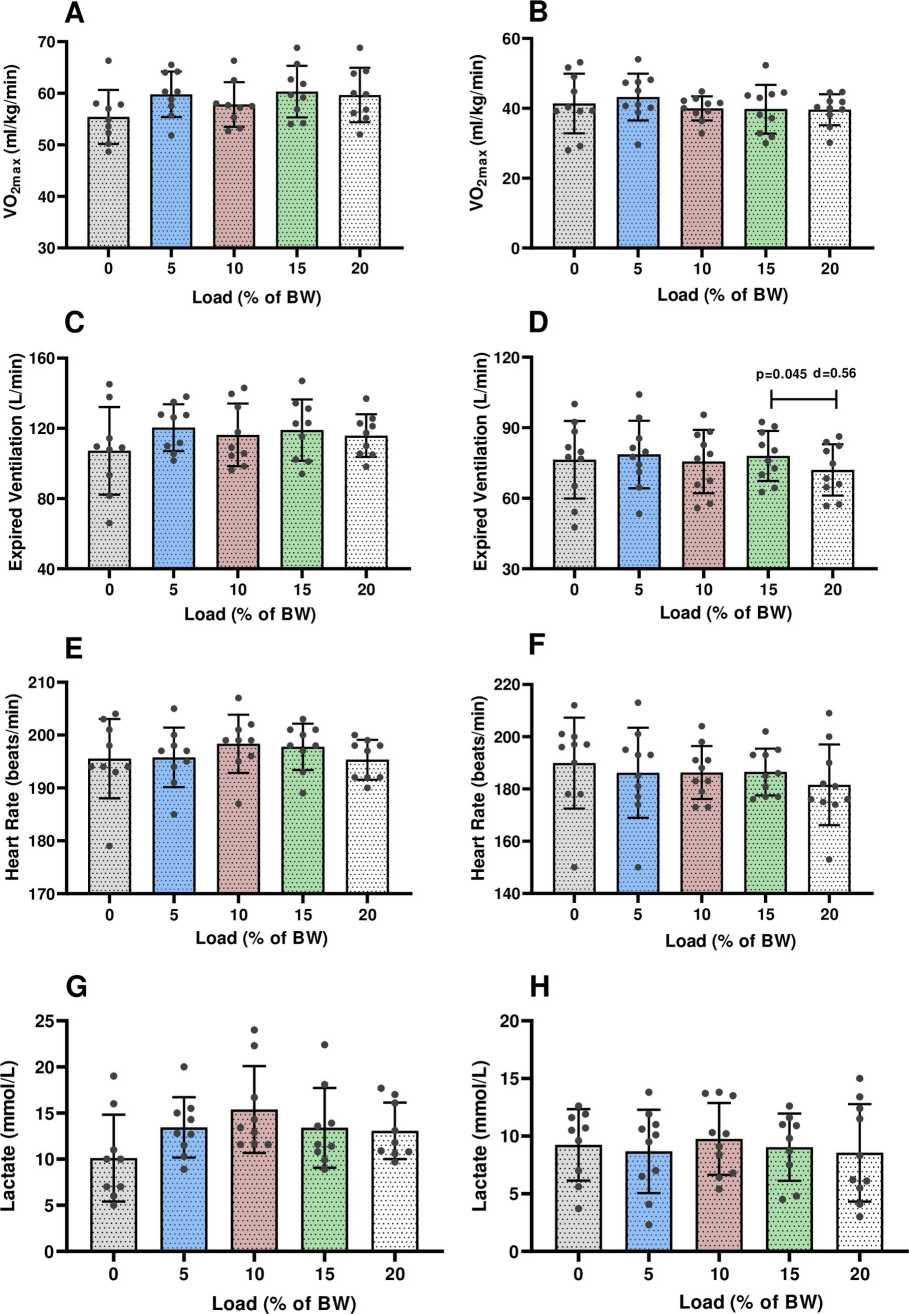
Mean and standard deviation of $\dot{V}O_{2\max}$ in (A) males and females (B), expired ventilation in males (C) and females (D), heart rate in males (E) and females (F) and lactate in males (G) and females (H) at different loads during the GXTs. BW, body weight; $\dot{V}O_{2\max}$, maximal oxygen uptake.

The ANOVA results indicate that there was no difference in $\dot{V}O_{2\max}$ (ml·kg^-1^·min^-1^) among the loading conditions in either group (p>0.05) (Fig 3A and 3B) suggesting that all $\dot{V}O_{2\max}$ remained similar during the GXTs in load and unloaded conditions. Maximal HR (Fig 3E and 3F) and blood lactate concentration (Fig 3G and 3H) remained unchanged between the unloaded and loaded conditions for both sexes (p>0.05) (S2–S4 Tables). Only female subjects showed reduced $\dot{V}E$ with 20% load carriage compared to 15% (Fig 3D) (15% BW = 77.9 ± 10.5 L/min vs. 20% BW = 72.0 ± 10.9 L/min; p = 0.045) (d = 0.56), while $\dot{V}E$ (Fig 3C and 3D) was similar at the other load carriages for both sexes (p>0.05).

## Discussion

The major question in the present study was whether or not the same or similar values for $\dot{V}O_{2\max}$ can be measured in the GXTs test with and without load carriage. The obtained findings showed that (1) the GXTs in load and unloaded conditions similarly measure $\dot{V}O_{2\max}$ in young males and females and (2) the GXTs under load measures $\dot{V}O_{2\max}$ in less time compared to the unloaded GXT protocol. Our finding shows that no matter the load % used during GXT, $\dot{V}O_{2\max}$, but not total exercise time, remains the same. Despite load carriage that substantially alters energy expenditure throughout the course of an incremental test, the endpoint is the attainment of the $\dot{V}O_{2\max}$. Therefore, researchers should not worry as to whether a given load can influence the capacity of individuals to attain $\dot{V}O_{2\max}$.

$\dot{V}O_{2\max}$ is accepted to be a strong predictor of endurance performance [44] and cardiovascular health in clinical patients [2, 45, 46]. The measure has a history dating back to 1923 and to the pioneering work of Hill and Lupton [39, 47]. Subsequent testing of $\dot{V}O_{2\max}$ was characterized by intermittent exercise protocols that were distributed over several days [48]. Despite additional research on protocols [49, 50], the ideal protocol duration to support a correct $\dot{V}O_{2\max}$ measurement was not addressed until the hallmark study of Buchfuhrer [10] in 1983. In the following years, different protocols were used to accurately measure $\dot{V}O_{2\max}$ [51–53] and GXT protocol has been one of the most widely used protocol in the basic and applied physiological sciences [54]. Here we found that the GXT with load carriage weighting to 15% and 20% BW decreased the GXT duration, compared to the unloaded GXT. These findings are in agreement with previous reports documenting reduced test duration (40% - 87.5%) with 25-kg backpack [22] and with 45-kg thoracic load carriage [19] during exercise testing on treadmill. The authors of these two studies also reported reduced $\dot{V}O_{2\mathrm{peak}}$ (3.5% - 5.4%) once the GXT was performed with 25-kg backpack and 45-kg thoracic load carriage load [19, 22]. However, $\dot{V}O_{2\max}$ remained unchanged in load and unloaded conditions during the GXTs in this study, which is in line with previous studies that reported no changes in $\dot{V}O_{2\max}$ with loads of 7%, 15%, 30% BW in comparison to the unloaded condition [24, 55, 56]. These discrepancies in $\dot{V}O_{2\max}$ are usually attributed to methodological differences (testing techniques, load carriages applied, subjects group (adolescent, adult, obese, healthy), physical activity level of the subjects included, variations in the load-carriage task modalities), motivational factors, different exercise modalities (walking, running, cycling). It is well-documented that heavy load carriage during exercise substantially increases the oxygen demand and lowers the energy available for locomotion [57], resulting in the same $\dot{V}O_{2\max}$. Besides, increased energy expenditure is required to normalize the body centre of mass under the load carriages [15], which plays an essential role in the physiological muscular activity of the arm and shoulder carrying a backpack load. Also, there would be a linear increase in metabolic energy expenditure proportionately with the load carried at a given speed (an added load of 30% BW produced a 10% increase in $\dot{V}O_{2}$; an added load of 40% BW produced a 20% increase in $\dot{V}O_{2}$) [16, 56, 58–60], suggesting that a significant load carriage increases energy cost and energy expenditure [61–63]. We also observed no difference in the $\dot{V}E$, blood lactate or maximal HR between the unloaded and loaded conditions, that are in agreement with previous studies [17, 23, 25]. In contrast, Phillips et al. reported the reductions in peak physiological responses and increased the exercise ventilatory requirement for oxygen with 45-kg thoracic loading compared to the unloaded condition [19]. Similarly, Wang et al. documented increased $\dot{V}E$ in young healthy males who performed exercise test under external chest loading [57]. These findings suggest that the loading conditions used in the current study may not provide enough potent stimulus to cause to the discomfort and cardiovascular strain during the load carriage task. It is worth noting that external load can cause physical fatigue, once work intensity is higher than 50% of maximal work capacity [64] due to altered locomotion biomechanics which in turn lead to the increase in energy cost. Therefore, carrying loads during GXTs should be used with some caution especially when applied to heavy loads. As well-documented, blood lactate concentration during exercise reflects the adaptive state of the muscle [39]. Consequently, the oxygen uptake at a given blood lactate concentration increases in response to regular training program [39, 65]. On the other hand, during GXT tests, increased adenosine triphosphate demand by the exercising muscles triggers glycolysis and pyruvate accumulation results in increased blood lactate levels. As reported in the results, blood lactate concentration measured immediately after the GXTs was similar, suggesting that similar physiological stress was elicited following the GXTs performed under different load carriages, resulting in similar blood lactate concentration.

In addition, exercise professionals should seek the exercise protocols not lasting too long by creating personalized incremental exercise protocol, or using validated population-specific standardized GXT protocol. Moreover, the weight of the loads applied during GXT must be calculated according to the BW of the subjects so that the backpacks are not too heavy to be carried. Otherwise, the subject will not be able to tolerate the test and will interrupt the test without meeting the test termination criteria, especially plateauing of $\dot{V}O_{2}$ while increasing work rate.

A few limitations of the study must be addressed here. Firstly, this investigation was completed with young, healthy males and females and as such, attempts to generalize to other demographics should be undertaken with appropriate caution. Secondly, we did not control the possible effects of the follicular phase and hormones such as progesterone and estrogen on the physiological variables in female subjects, known to affect athletic performance [66], that has recently been shown not to be the case [67, 68]. We also recruited healthy and young subjects who were relatively unpracticed with load carriage on the treadmill, so we couldn’t increase the load carriage more than 20% BW that limited our findings. Further studies are needed to investigate the physiological effects of load conditions heavier than 20% BW during the exercise testing in different population. Additionally, we utilized an identical backpack of which packing was fitted similarly by the same experimenter. However, while the selected backpack was an appropriate size for all subjects, a ‘‘perfect” fit could not be provided for all. Another limitation associated with this study is that increase in type I error rate across the reported statistical analyses was not controlled since the $\dot{V}O_{2\max}$ was the primary outcome, while the other parameters measured ($\dot{V}E$, HR, blood lactate) were considered as the secondary outcomes. Overall, we certainly encourage further studies that will consider correcting for the type I error.

## Conclusion

The main findings of this study show that the GXT performed with or without load similarly measures $\dot{V}O_{2\max}$ for both sexes. Also, the GXTs performed with 15% and 20% BW load can reduce GXT test duration.

## Supporting information

S1 Table Modified Bruce protocol. (DOCX)

S2 TableMean and standard deviation of $\dot{V}O_{2max},\;maximal\;HR,\;\dot{V}E$, post-test lactate, maximal running speed and test duration in male and female subjects.(DOCX)

S3 Table Statistical results of the variables of male subjects. (DOCX)

S4 Table Statistical results of the variables of female subjects. (DOCX)
