# Supplementary material for: Graded exercise test with or without load carriage similarly measures maximal oxygen uptake in young males and females
Source: PLoS One. 2021 Feb 1;16(2):e0246303. doi: 10.1371/journal.pone.0246303 (PMC7850508; doi:10.1371/journal.pone.0246303)
Supplement: S1 Table — (DOCX) [file pone.0246303.s001.docx]

**S1 Table.** Modified Bruce Protocol

| **Stage** | **Speed (km h^-1^)** | **Incline (%)** | **Duration (min)** | **Duration total (min)** |
| --- | --- | --- | --- | --- |
| 0 | 2.7 | 0 | 3 | 3 |
| 0.5 | 2.7 | 5 | 3 | 6 |
| 1 | 2.7 | 10 | 3 | 9 |
| 2 | 4 | 12 | 3 | 12 |
| 3 | 5.4 | 14 | 3 | 15 |
| 4 | 6.7 | 15 | 3 | 18 |
| 5 | 8 | 15 | 3 | 21 |
| 6 | 8.8 | 15 | 3 | 24 |
| 7 | 9.6 | 15 | 3 | 27 |
