# Supplementary material for: Graded exercise test with or without load carriage similarly measures maximal oxygen uptake in young males and females
Source: PLoS One. 2021 Feb 1;16(2):e0246303. doi: 10.1371/journal.pone.0246303 (PMC7850508; doi:10.1371/journal.pone.0246303)
Supplement: S2 Table — (DOCX) [file pone.0246303.s002.docx]

| **S2 Table.** Mean and standard deviation of VO_2max_, maximal HR, VE, post-test lactate, maximal running speed and test duration in male and female subjects | | | | | | | | | | |
| --- | --- | --- | --- | --- | --- | --- | --- | --- | --- | --- |
|  | **Male** | | | | | **Female** | | | | |
|  | **Unloaded** | **5% BW** | **10% BW** | **15% BW** | **20% BW** | **Unloaded** | **5% BW** | **10% BW** | **15% BW** | **20% BW** |
| $\dot{\mathbf{V}}$**O_2max_** (ml/kg/min) | 55.4±5.2 | 59.7±4.4 | 57.8±4.3 | 60.3±5.0 | 59.6±5.2 | 41.3±8.5 | 43.2±6.7 | 39.6±3.4 | 39.7±6.9 | 39.6±4.4 |
| **Maximal HR** (beat/min) | 195.5±7.5 | 195.7±5.6 | 198.3±5.5 | 197.7±4.3 | 195.3±3.7 | 189.9±17.4 | 186.2±17.2 | 186.2±10.1 | 186.5±8.9 | 181.6±15.4 |
| $\dot{\mathbf{V}}$**E**  (L/min) | 107.2±24.9 | 120.4±13.3 | 116.2±17.8 | 119.0±17.4 | 115.8±12.1 | 76.3±16.4 | 78.6±14.3 | 75.6±13.4 | 77.9±10.5 | 72.0±10.9 |
| **Post-test lactate** (mmol/L) | 10.6±4.6 | 13.4±3.2 | 15.3±4.7 | 13.4±4.3 | 13.0±3.1 | 10.2±4.3 | 8.6±3.6 | 9.7±3.1 | 10.5±5.5 | 8.5±4.2 |
| **Maximal running** **speed** (km/h) | 14.8±1.1 | 15.3±1.0 | 14.6±0.6 | 14.0±1.0 | 13.5±0.7 | 13.5±1.0 | 12.6±0.7 | 12.1±1.1 | 11.5±1.0 | 11.1±1.1 |
| **Test duration** (min) | 22.11±2.0 | 22.7±1.6 | 21.6±1.2 | 20.5±1.5 | 19.6±1.1 | 18.1±3.6 | 17.4±2.9 | 16.7±1.5 | 15.9±1.6 | 15.2±2.3 |

BW; body weight, $\dot{V}$O_2max_; maximal oxygen uptake, HR; heart rate, $\dot{V}$E; expired ventilation, W; watt. Mean ± SD
