# Supplementary material for: Graded exercise test with or without load carriage similarly measures maximal oxygen uptake in young males and females
Source: PLoS One. 2021 Feb 1;16(2):e0246303. doi: 10.1371/journal.pone.0246303 (PMC7850508; doi:10.1371/journal.pone.0246303)
Supplement: S3 Table — (DOCX) [file pone.0246303.s003.docx]

**S3 Table.** Statistical results of the variables of male subjects

|  |  | $\dot{\mathbf{V}}$**O_2max_** | | | **Maximal HR** | | | **Expired Ventilation** | | | **Post-test blood lactate** | | |
| --- | --- | --- | --- | --- | --- | --- | --- | --- | --- | --- | --- | --- | --- |
|  |  | **Sig** | **95%**  **Confidence Interval** | | **Sig** | **95%**  **Confidence Interval** | | **Sig** | **95%**  **Confidence Interval** | | **Sig** | **95%**  **Confidence Interval** | |
|  |  |  | **Lower**  **Bound** | **Upper Bound** |  | **Lower**  **Bound** | **Upper**  **Bound** |  | **Lower**  **Bound** | **Upper**  **Bound** |  | **Lower**  **Bound** | **Upper**  **Bound** |
| **Unloaded** | **5% BW** | .364 | -11.071 | 2.311 | 1.000 | -4.385 | 11.785 | .368 | -33.394 | 7.016 | .466 | -7.452 | 1.786 |
|  | **10% BW** | 1.000 | -8.459 | 3.659 | 1.000 | -10.944 | 18.144 | .573 | -24.724 | 6.593 | .272 | -11.565 | 2.009 |
|  | **15% BW** | .100 | -10.512 | .700 | 1.000 | -10.690 | 17.490 | .182 | -27.121 | 3.494 | .174 | -6.367 | .790 |
|  | **20% BW** | .272 | -10.290 | 1.790 | .174 | -2.238 | 18.838 | 1.000 | -31.677 | 14.366 | 1.000 | -8.479 | 3.545 |
| **5% BW** | **Unloaded** | .364 | -2.311 | 11.071 | 1.000 | -11.785 | 4.385 | .368 | -7.016 | 33.394 | .466 | -1.786 | 7.452 |
|  | **10% BW** | .489 | -1.291 | 5.251 | 1.000 | -12.404 | 12.204 | 1.000 | -10.301 | 18.547 | 1.000 | -9.528 | 5.640 |
|  | **15% BW** | 1.000 | -5.088 | 4.037 | 1.000 | -12.525 | 11.925 | 1.000 | -12.135 | 14.886 | 1.000 | -5.768 | 5.857 |
|  | **20% BW** | 1.000 | -3.224 | 3.484 | .214 | -1.505 | 10.705 | 1.000 | -10.106 | 19.173 | 1.000 | -4.315 | 5.048 |
| **10% BW** | **Unloaded** | 1.000 | -3.659 | 8.459 | 1.000 | -18.144 | 10.944 | .573 | -6.593 | 24.724 | .272 | -2.009 | 11.565 |
|  | **5% BW** | .489 | -5.251 | 1.291 | 1.000 | -12.204 | 12.404 | 1.000 | -18.547 | 10.301 | 1.000 | -5.640 | 9.528 |
|  | **15% BW** | 1.000 | -7.897 | 2.886 | 1.000 | -4.081 | 3.681 | 1.000 | -13.473 | 7.977 | .650 | -1.577 | 5.555 |
|  | **20% BW** | 1.000 | -5.686 | 1.986 | 1.000 | -5.722 | 15.122 | 1.000 | -14.840 | 15.660 | 1.000 | -4.616 | 9.238 |
| **15% BW** | **Unloaded** | .100 | -.700 | 10.512 | 1.000 | -17.490 | 10.690 | .182 | -3.494 | 27.121 | .174 | -.790 | 6.367 |
|  | **5% BW** | 1.000 | -4.037 | 5.088 | 1.000 | -11.925 | 12.525 | 1.000 | -14.886 | 12.135 | 1.000 | -5.857 | 5.768 |
|  | **10% BW** | 1.000 | -2.886 | 7.897 | 1.000 | -3.681 | 4.081 | 1.000 | -7.977 | 13.473 | .650 | -5.555 | 1.577 |
|  | **20% BW** | 1.000 | -5.661 | 6.973 | 1.000 | -5.842 | 15.642 | 1.000 | -6.694 | 13.009 | 1.000 | -5.618 | 6.262 |
| **20% BW** | **Unloaded** | .272 | -1.790 | 10.290 | .174 | -18.838 | 2.238 | 1.000 | -14.366 | 31.677 | 1.000 | -3.545 | 8.479 |
|  | **5% BW** | 1.000 | -3.484 | 3.224 | .214 | -10.705 | 1.505 | 1.000 | -19.173 | 10.106 | 1.000 | -5.048 | 4.315 |
|  | **10% BW** | 1.000 | -1.986 | 5.686 | 1.000 | -15.122 | 5.722 | 1.000 | -15.660 | 14.840 | 1.000 | -9.238 | 4.616 |
|  | **15% BW** | 1.000 | -6.973 | 5.661 | 1.000 | -15.642 | 5.842 | 1.000 | -13.009 | 6.694 | 1.000 | -6.262 | 5.618 |

BW; body weight, $\dot{V}$O_2max_; maximal oxygen uptake
