# Supplementary material for: Graded exercise test with or without load carriage similarly measures maximal oxygen uptake in young males and females
Source: PLoS One. 2021 Feb 1;16(2):e0246303. doi: 10.1371/journal.pone.0246303 (PMC7850508; doi:10.1371/journal.pone.0246303)
Supplement: S4 Table — (DOCX) [file pone.0246303.s004.docx]

**S4 Table.** Statistical results of the variables of female subjects

|  |  | $\dot{\mathbf{V}}$**O_2max_** | | | **Maximal HR** | | | **Expired Ventilation** | | | **Post-test blood lactate** | | |
| --- | --- | --- | --- | --- | --- | --- | --- | --- | --- | --- | --- | --- | --- |
|  |  | **Sig** | **95%**  **Confidence Interval** | | **Sig** | **95%**  **Confidence Interval** | | **Sig** | **95%**  **Confidence Interval** | | **Sig** | **95%**  **Confidence Interval** | |
|  |  |  | **Lower**  **Bound** | **Upper Bound** |  | **Lower**  **Bound** | **Upper**  **Bound** |  | **Lower**  **Bound** | **Upper**  **Bound** |  | **Lower**  **Bound** | **Upper**  **Bound** |
| **Unloaded** | **5% BW** | 1.000 | -7.523 | 3.837 | 1.000 | -4.385 | 11.785 | 1.000 | -15.070 | 10.548 | .173 | -.425 | 3.605 |
|  | **10% BW** | 1.000 | -8.191 | 11.029 | 1.000 | -10.944 | 18.144 | 1.000 | -18.907 | 20.431 | 1.000 | -2.351 | 3.371 |
|  | **15% BW** | 1.000 | -4.257 | 7.525 | 1.000 | -10.690 | 17.490 | 1.000 | -18.114 | 14.874 | 1.000 | -6.223 | 5.623 |
|  | **20% BW** | 1.000 | -6.655 | 10.221 | .174 | -2.238 | 18.838 | 1.000 | -12.276 | 20.870 | 1.000 | -2.224 | 5.664 |
| **5% BW** | **Unloaded** | 1.000 | -3.837 | 7.523 | 1.000 | -11.785 | 4.385 | 1.000 | -10.548 | 15.070 | .173 | -3.605 | .425 |
|  | **10% BW** | 1.000 | -5.309 | 11.833 | 1.000 | -12.404 | 12.204 | 1.000 | -13.196 | 19.242 | .986 | -3.243 | 1.083 |
|  | **15% BW** | 1.000 | -4.641 | 11.595 | 1.000 | -12.525 | 11.925 | 1.000 | -13.038 | 14.320 | 1.000 | -7.258 | 3.478 |
|  | **20% BW** | .926 | -3.486 | 10.738 | .214 | -1.505 | 10.705 | .871 | -6.045 | 19.161 | 1.000 | -3.707 | 3.967 |
| **10% BW** | **Unloaded** | 1.000 | -11.029 | 8.191 | 1.000 | -18.144 | 10.944 | 1.000 | -20.431 | 18.907 | 1.000 | -3.371 | 2.351 |
|  | **5% BW** | 1.000 | -11.833 | 5.309 | 1.000 | -12.204 | 12.404 | 1.000 | -19.242 | 13.196 | .986 | -1.083 | 3.243 |
|  | **15% BW** | 1.000 | -6.878 | 7.308 | 1.000 | -4.081 | 3.681 | 1.000 | -14.706 | 9.942 | 1.000 | -7.305 | 5.685 |
|  | **20% BW** | 1.000 | -4.262 | 4.990 | 1.000 | -5.722 | 15.122 | 1.000 | -5.591 | 12.661 | 1.000 | -2.014 | 4.434 |
| **15% BW** | **Unloaded** | 1.000 | -7.525 | 4.257 | 1.000 | -17.490 | 10.690 | 1.000 | -14.874 | 18.114 | 1.000 | -5.623 | 6.223 |
|  | **5% BW** | 1.000 | -11.595 | 4.641 | 1.000 | -11.925 | 12.525 | 1.000 | -14.320 | 13.038 | 1.000 | -3.478 | 7.258 |
|  | **10% BW** | 1.000 | -7.308 | 6.878 | 1.000 | -3.681 | 4.081 | 1.000 | -9.942 | 14.706 | 1.000 | -5.685 | 7.305 |
|  | **20% BW** | 1.000 | -6.121 | 6.419 | 1.000 | -5.842 | 15.642 | .045 | .101 | 11.733 | 1.000 | -5.478 | 9.518 |
| **20% BW** | **Unloaded** | 1.000 | -10.221 | 6.655 | .174 | -18.838 | 2.238 | 1.000 | -20.870 | 12.276 | 1.000 | -5.664 | 2.224 |
|  | **5% BW** | .926 | -10.738 | 3.486 | .214 | -10.705 | 1.505 | .871 | -19.161 | 6.045 | 1.000 | -3.967 | 3.707 |
|  | **10% BW** | 1.000 | -4.990 | 4.262 | 1.000 | -15.122 | 5.722 | 1.000 | -12.661 | 5.591 | 1.000 | -4.434 | 2.014 |
|  | **15% BW** | 1.000 | -6.419 | 6.121 | 1.000 | -15.642 | 5.842 | .045 | -11.733 | -.101 | 1.000 | -9.518 | 5.478 |

BW; body weight, $\dot{V}$O_2max_; maximal oxygen uptake
